# Psychotic disorders in young patients with Prader-Willi syndrome: A case report and literature review

**DOI:** 10.1192/j.eurpsy.2023.853

**Published:** 2023-07-19

**Authors:** O. De Juan Viladegut, M. Llobet Farré, H. Andreu Gracia, L. Bueno Sanya, L. Olivier Mayorga, A. Morer Liñan, L. Lázaro García, A. E. Ortiz García

**Affiliations:** 1 Department of Psychiatry and Psychology, Institute of Neurosciences.; 2 Department of Child and Adolescent Psychiatry and Psychology, Institute of Neurosciences., Hospital Clínic de Barcelona, Barcelona, Spain

## Abstract

**Introduction:**

Prader-Willi syndrome (PWS) is a genetic disorder with an estimated prevalence of 1:25,000. PWS results from defective gene expression on the paternal copy of chromosome 15. In 70% of the cases it is a deletion that means that part of the paternal chromosome 15 is missing. Maternal uniparental disomy (mUPD) is present in 25% of cases. Typical clinical features of PWS are dysmorphism, hypotonia, hyperphagia, hypogonadism and developmental delay. In addition, the syndrome is accompanied by various psychiatric symptoms that are often insufficiently known within the psychiatric field. Regarding the relationship between PWS and schizophrenia spectrum disorders (SSDs), individuals with mUPD appear to have a 3 to 4 times higher risk of psychotic symptoms than those with the deletion subtype. Psychotic episodes have an atypical presentation with recurrent episodes of confusion and rapidly fluctuating psychotic and mood symptoms.

**Objectives:**

To describe an unusual clinical case in order to determine the management regarding clinical approach, and provide an overview of psychotic episodes in patients with PWS for the general practitioner with the most up-to-date information on workup and management.

**Methods:**

We report a case involving a 13-year-old woman with PWS (mUPD of chromosome 15) and mild intellectual disability (IQs 59), who presented psychotic symptomatology in the form of disorganized behavior, delusional ideation, auditory hallucinations, self-referentiality and suspicion. Parents reported that these symptoms started two days prior the day of consultation. No environmentals stressors were identified and no recent treatment changes were made. Patient’s medication consists in 150 mg sertraline per day due to anxiety control and aid in emotional and behavioral regulation.

**Results:**

Given the diagnostic approach of a psychotic episode (PE) in a patient with PWS, it was decided to offer 0.5mg risperidone per day, in an increasing pattern until reaching a final dose of 1.25 mg per day, presenting a global remission of the psychotic symptomatology.

Recommendations for patients with PWS presenting PE are based upon systematic reviews. Patients with PWS, especially mUPD subjects, are at risk for SSDs and mood disorders. Antipsychotics (APs) are the gold standard in the treatment of SSDs, and some authors have suggested that APs protect patients with previous psychotic symptoms from relapse. It is unknown whether there is a protective effect of APs in mUPD patients who have not previously exhibited psychotic signs.

**Conclusions:**

PWS represents a good example of a genetic disease with behavioral and psychiatric symptoms that may be challenging to treat with psychotropic medications. For a better understanding of psychiatric problems in adults with PWS, longitudinal studies with careful and standardized follow-up of psychiatric symptoms in PWS are necessary.

**Disclosure of Interest:**

None Declared

